# Editorial: A new science of suffering, the wisdom of the soul, and the new behavioral economics of happiness: towards a general theory of well-being

**DOI:** 10.3389/fpsyg.2023.1280613

**Published:** 2023-09-21

**Authors:** Paul T. P. Wong, Lok Sang Ho, Claude-Hélène Mayer, Fan Yang, Richard G. Cowden

**Affiliations:** ^1^Department of Psychology, Trent University, Peterborough, ON, Canada; ^2^STEAM Education and Research Center, Lingnan University, Tuen Mun, Hong Kong SAR, China; ^3^Department of Industrial Psychology and People Management, University of Johannesburg, Johannesburg, South Africa; ^4^Department of Psychology, The University of Chicago, Chicago, IL, United States; ^5^Human Flourishing Program, Harvard University, Cambridge, MA, United States

**Keywords:** existential positive psychology, flourishing, happiness, life intelligence, suffering, well-being

This Research Topic is designed to extend the theme of existential positive psychology (EPP) to new research areas. Theoretically, it is oriented toward a general theory of global well-being, which incorporates three pillars of EPP (Wong et al., 2022):

(1) the existential universals of suffering, ultimate concerns, and the deep-seated human yearning for meaning, social connection, and spirituality;

(2) unique expressions and experiences of existential universals in different seasons of life and different cultures; and

(3) personal transformation through suffering.

A general theory of well-being needs to cover the complete spectrum of existential well-being, which includes not only different facets of personhood but also wide-ranging dynamics of nature and culture that affect human existence, such as globalization, climate change, ecology, and the mysterious invisible forces capable of impacting well-being. It must be capable of integrating the bright and dark sides of life, as well as the unknown forces that may benefit or threaten humanity.

## Shifting to a new science of suffering

A new science of suffering (also known as positive psychology of suffering) is needed to better understand (1) different kinds of suffering (e.g., necessary vs. unnecessary suffering) and (2) the bidirectionality of suffering (i.e., the conditions under which suffering can either degrade or strengthen us). This new science of suffering is essential for creating a more complete picture of human flourishing, just as the science of pain and disease control is essential for physical health and medical science.

Melios et al. provide one of the latest multinational empirical studies on low subjective well-being by leveraging cross-sectional data from the Gallup World Poll. Although individual-level factors had the greatest explanatory power, evidence of interactions between individual and country-level factors on subjective well-being support the idea that human flourishing is shaped by a complex system involving people and the places in which they live (Counted et al., 2021; VanderWeele et al., 2022).

Fayard and Mayer's qualitative study indicated that young male university graduates understand the challenges of transitioning to the workplace and are able to transform their stressful experiences into a salutogenic process. Their work represents a paradigm shift from a pathogenic approach toward a positive health paradigm in which an individual's position on the continuum from health to disease is determined by the interaction of environmental threats, their degree of resistance, and the strength of their sense of coherence.

Kaftanski and Hanson's conceptual article forms part of the emerging trend of flipping the common narrative that suffering is wholly an impediment to human well-being. Consistent with recent theoretical (e.g., Wong et al., 2022) and empirical literature (e.g., Wilkinson et al., 2023), the authors recognize that conceptions of well-being typically overlook suffering or assume an unrealistic version of human life in which suffering is nonexistent.

Sease et al.'s perspective article focuses on existential isolation, which is a special case of existential suffering (Wong, 2015). Based on their review of relevant literature, they theorize that existential isolation could thwart therapeutic interventions in justice settings because people involved in the justice system may feel more disjointed from their counselors and peers.

Güven and Arslan explore EPP by studying themes of suffering and happiness in Turkish folk poetry. Their research shows that suffering is an inescapable part of human life, but it can also be source for building resilience. These findings support the notion of transforming suffering for an adaptive purpose (Ho et al., 2022; VanderWeele et al., 2023), and suggest that suffering has the potential to promote growth and contribute to mature happiness.

Wong and Laird's perspective article explores the universality and complexity of human suffering. They present some possible ways of classifying suffering in everyday life as well as in the clinical setting, while acknowledging the difficulty of developing a complete taxonomy of suffering.

## Advancing a framework of existential intelligence

Since a general theory of well-being deals with big questions about human nature and human existence, existential intelligence (also known as existential thinking or life intelligence) plays a major role in the prevention and transformation of suffering. Existential intelligence involves having the necessary existential wisdom to navigate adverse situations in ways that lead to a meaningful and honorable life, and it may be one of the most important capacities for dealing with the complex questions related to good and evil, happiness and suffering, as well as life and death. For example, in situations where there is a need to balance conflicting values and demands, a person must consult their conscience and wisdom of the soul to decide on the best course of action.

In Ge and Yang's perspective article, they explore some possible mechanisms by which self-transcendence enables people to endure and transcend suffering. From an examination of the empirical literature, they propose that self-transcendence may support endurance of suffering at three psychological levels: (1) self-transcendent experiences (affect), (2) self-transcendent thinking (cognition), and (3) need for self-transcendence (motivation).

Lau et al.'s brief report investigates the relationship between mindfulness, stress, savoring beliefs, and life satisfaction in a cross-sectional sample of Hong Kong adolescents during the COVID-19 pandemic. They found that mindfulness is related to lower stress and greater life satisfaction and savoring beliefs.

In Kam and Bellehumeur's perspective article, they argue that deeper levels of unconsciousness are needed to adaptively cope with the uncomfortable experience of the ambiguous coexistence of opposites. Their work is comparable to ideas concerning wisdom of the soul or life intelligence, which explicate the importance of navigating and resolving the paradoxes of human existence through transcending opposites (Wong et al., 2021).

Horikoshi's perspective article focuses on the positive psychology of challenge. He argues that studying activities and processes involving challenges can provide insights into dialectical integration of opposites because the concept of challenge encompasses both positive and negative elements.

Lau and Tov used experimental data from a sample of Singaporean university students to explore whether meaning-making strategies facilitate adaptive processing of daily negative experiences. They found that positive reappraisal and self-distancing affected situational meaning, but that the circumstances under which these strategies supported meaning-making varied. This work emphasizes the importance of wisely selecting and applying different coping strategies to effectively make meaning out of negative life events.

Rajkumar's brief research report uses data from the 2021 World Happiness Report to explore the relationship between culture and self-reported happiness in 78 countries before (2017–2019) and during (2020–2021) the COVID-19 pandemic. The findings provide some support for the importance of studying cultural differences in existential universals, such as happiness and suffering.

Han et al. used repeated cross-sectional data from Chinese adolescents to examine group orientation and mental health before (2019) and during the COVID-19 pandemic (2021). Their findings demonstrated the protective value of group orientation in transcending egotistic concerns during the COVID-19 pandemic, and that contextual factors may influence the protective benefits of group orientation on mental health.

In their original research article, Liu et al. use two waves of longitudinal data from a sample of Chinese adolescents to investigate whether the relationship between self-transcendence values and emotional adjustment is mediated by emotion regulation. Their findings shed light on potential avenues to support adolescents' emotional adjustment, as well as provide further evidence on the benefits of self-transcendence.

## Developing a new behavioral economics of happiness

The new behavioral economics of happiness does not presume that human beings are well-informed rational decision-makers capable of choosing what is in their best interest. Instead, it recognizes that rational choices are affected by three human limitations: (1) humans often have flawed perceptions of reality, including what they really want in order to be happy; (2) universal human challenges, such as difficulties exercising self-control and a lack of self-understanding; and (3) the tendency for humans to choose immediate self-gratification rather than the long-term gain of something meaningful and of enduring value. It recognizes that true flourishing is only possible when people are awakened to the existential truth of overcoming their dark side and “inner demons” to become who they are meant to be.

In their opinion article, Mead et al. suggest that personal development progress must be reinforced by a commitment to making systemic changes that allow for new “ecological economics” to emerge in post-growth societies. The authors suggest that the new era of Symbiocene has much potential for developing evidenced-based approaches that could shape government policies and transform societies.

Tweed et al.'s conceptual article draws on ideas from Martin Buber to touch on the core issue of developing a new model of behavioral economics. Rather than viewing people as instruments to be used for advancing self-interests, this model advocates treating people as human beings (i.e., as ends in and of themselves rather than as means to an end). By simply changing our views and attitudes toward others, we can become more just, authentic, and compassionate in our interactions with others.

## Conclusion

This Research Topic draws attention to some key blind spots in research on well-being, including notions that (1) flourishing necessarily involves the dialectic integration of positives and negatives; (2) ideal happiness is more about inner peace, balance, and harmony in the midst of adversity and hardship than attaining maximum happiness; (3) triumphing over suffering requires having a courageous stance toward one's fate and making conscionable choices despite the constraints imposed by one's circumstances; and (4) the science of human flourishing requires a delicate balance between studying existential universals, the particularities of each culture, and the unique experiences of each person (Arslan and Wong, 2021; Wong et al., 2021; Wong and Cowden, 2022; Cowden et al., 2023). These points are at the heart of EPP's research agenda and align with a dialectic general theory of well-being, which is constituted by the integration of human agency and divine support, noble idealism and brutal realism, and ancient wisdom in the humanities and the scientific research of contemporary psychology.

## Author contributions

PW: Conceptualization, Writing—original draft, Writing—review and editing. LH: Writing—review and editing. C-HM: Writing—review and editing. FY: Writing—review and editing. RC: Conceptualization, Writing—review and editing.
